# Analysis of the Growth and Metabolites of a Pyruvate Dehydrogenase Complex-Deficient *Klebsiella pneumoniae* Mutant in a Glycerol-Based Medium

**DOI:** 10.4014/jmb.1801.01045

**Published:** 2018-05-11

**Authors:** Danfeng Xu, Zongxiao Jia, Lijuan Zhang, Shuilin Fu, Heng Gong

**Affiliations:** State Key Laboratory of Bioreactor Engineering, East China University of Science and Technology, Shanghai 200237, P.R. China

**Keywords:** 1, 3-propanediol, glycerol, *Klebsiella pneumoniae*, pyruvate dehydrogenase complex, pyruvate formate lyase

## Abstract

To determine the role of pyruvate dehydrogenase complex (PDHC) in *Klebsiella pneumoniae*, the growth and metabolism of PDHC-deficient mutant in glycerol-based medium were analyzed and compared with those of other strains. Under aerobic conditions, the PDHC activity was fourfold higher than that of pyruvate formate lyase (PFL), and blocking of PDHC caused severe growth defect and pyruvate accumulation, indicating that the carbon flux through pyruvate to acetyl coenzyme A mainly depended on PDHC. Under anaerobic conditions, although the PDHC activity was only 50% of that of PFL, blocking of PDHC resulted in more growth defect than blocking of PFL. Subsequently, combined with the requirement of CO_2_ and intracellular redox status, it was presumed that the critical role of PDHC was to provide NADH for the anaerobic growth of *K. pneumoniae*. This presumption was confirmed in the PDHC-deficient mutant by further blocking one of the formate dehydrogenases, FdnGHI. Besides, based on our data, it can also be suggested that an improvement in the carbon flux in the PFL-deficient mutant could be an effective strategy to construct highyielding 1,3-propanediol-producing *K. pneumoniae* strain.

## Introduction

With growing environmental issues and limited availability of fossil fuels, much interest has been focused on the utilization of biodiesel, one of the important biofuels produced by transesterification or ethanolysis of biological feedstock [1]. However, biodiesel industry also produces glycerol of approximately 10 wt.% of the total product [2]. Recently, owing to the rapid increase in biodiesel production, the output of glycerol has dramatically improved, decreasing the value of glycerol [3, 4]. Therefore, it is essential to find alternative uses for glycerol. The bioconversion of glycerol into 1,3-propanediol (1,3-PD) is attractive because 1,3-PD has high value and widespread use in polyester, cosmetic, and pharmaceutical industries [5, 6]. Many microorganisms can utilize glycerol to produce 1,3-PD, and among them, *Klebsiella pneumoniae* is the most extensively studied with proven superior ability to produce 1,3-PD [7, 8].

**Fig. 1:**
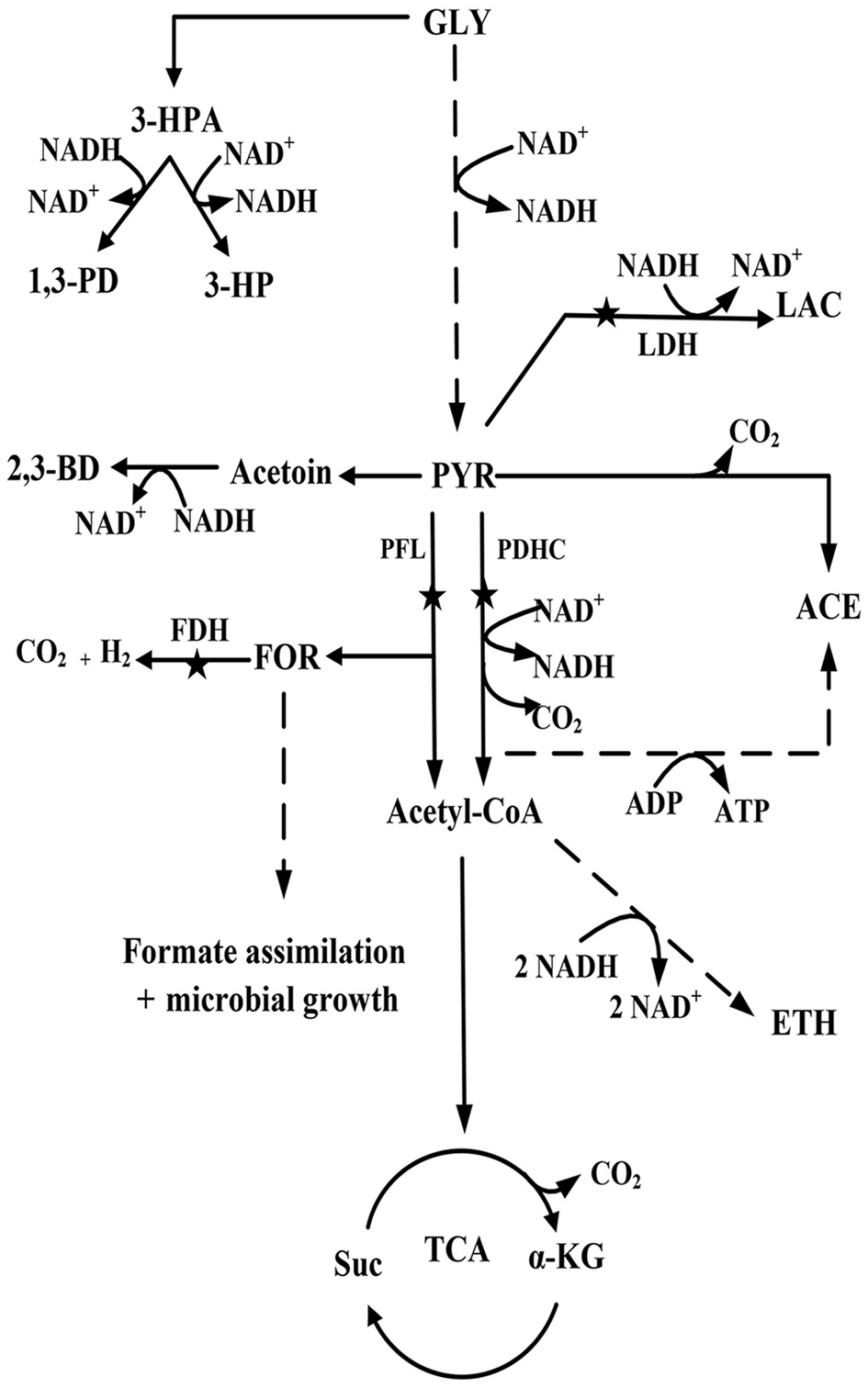
Metabolic pathways of glycerol metabolism in *K. pneumoniae*. GLY glycerol, 3-HPA 3-hydroxypropinaldehyde, 3-HP 3-hydroxypropionic, 1,3-PD 1,3-propanediol, 2,3-BD 2,3-butanediol, PYR pyruvate, LAC lactate, FOR formate, ACE acetate, ETH ethanol, SUC succinate, α-KG α-ketoglutarate, TCA tricarboxylic acids cycle, PDHC pyruvate dehydrogenase complex, PFL pyruvate formate lyase, FDH formate dehydrogenase, LDH lactate dehydrogenase. Stars indicate metabolic reactions that have been blocked by gene deletions.

The metabolic pathway of *K. pneumoniae* using glycerol as the sole carbon source is summarized in Fig. 1. In general, glycerol is converted through reductive and oxidative pathways, with the reductive branch leading to 3-hydroxypropionaldehyde formation and then 1,3-PD synthesis, while the oxidative branch providing the intermediate metabolite pyruvate and concomitantly generating the reducing power (NADH) for 1,3-PD synthesis [9, 10]. In the reductive branch, 3-hydroxypropionaldehyde can also be oxidized to 3-hydroxypropionic acid (3-HP). Accordingly, an increase in 3-HP improved NADH generation and further promoted 1,3-PD synthesis [11]. Nevertheless, pyruvate metabolism is the most important step affecting 1,3-PD synthesis, because the metabolites originating from pyruvate lead to the decrease in carbon flux and reduction in the ability to synthesize 1,3-PD. Therefore, various genetic modifications of *K. pneumoniae* have been investigated on pyruvate node, such as blocking of the flux to lactate, acetate, or 2,3-butanediol (2,3-BD), etc. [10, 12, 13]. However, only few reports have focused on the key route of pyruvate to acetyl coenzyme A (AcCoA).

In Enterobacteria, pyruvate is catabolized to AcCoA by pyruvate dehydrogenase complex (PDHC) or pyruvate formate lyase (PFL), which represents a switch point between respiratory and fermentative metabolism. In general, during aerobic respiration, pyruvate is converted to AcCoA, CO_2_, and NADH by PDHC, whereas during anaerobic fermentation, pyruvate is converted to AcCoA and formate by PFL [14-16]. However, evidences show that PDHC could function even under anaerobic conditions. In *Escherichia coli*, a significant flux through PDHC was observed, and the CO_2_ generated by PDHC was shown to be necessary for anaerobic cell growth [17]. It must be noted that 1,3-PD is a common anaerobic product during glycerol fermentation, and pyruvate catabolized by PDHC can generate NADH, which is beneficial for 1,3-PD production. Flux and enzyme analysis in *K. pneumoniae* has suggested that PDHC could function during anaerobic fermentation [18, 19], although the exact role of PDHC remains unclear.

In the present study, the growth and cell metabolism of PDHC-deficient mutant were analyzed in glycerol-based medium and compared with those of other mutants. It was found that blocking of PDHC resulted in severe growth defect of *K. pneumoniae* even under anaerobic condition. Furthermore, the roles of PDHC were determined, which were noted to be different from those in *E. coli* under anaerobic condition. Based on these data, a strategy for the construction of high-yielding 1,3-PD-producing *K. pneumoniae* strain was also discussed.

## Materials and Methods

### Strains, Plasmids, and Primers

The strains, plasmids, and primers used in this study are listed in Table 1. The lactate-deficient *K. pneumoniae* strain KG2 [11], derived from *K. pneumoniae* strain KG (CCTCC M2014574) by deleting *ldhA*, is a high-yielding 1,3-PD-producing strain, and was used as the parent strain in this study.

### Cultivation Conditions

The parent strain was precultured in test tubes containing 5 ml of Luria broth (10 g/l Difco tryptone, 5 g/l Difco yeast extract, and 10 g/l NaCl) for 10 h at 37°C and 200 rpm. Subsequently, for the assessment of mutants, flask batch cultures with 1% inoculum were accomplished at 37°C for 24 h with shaking at 300 and 200 rpm under aerobic and anaerobic conditions, respectively. For aerobic cultivation, 250-ml shake flasks containing 20 ml of the culture medium were covered with eight layers of gauze to allow air penetration. For anaerobic cultivation, 250-ml shake flasks containing 50 ml of the culture medium were plugged with a gas-impermeable rubber stopper, the air in the flasks was replaced with N_2_ gas before cultivation, and the flasks were subsequently plugged with a gas-impermeable rubber stopper. The culture medium (pH 7.0) contained 60 g/l glycerol, 1 g/l KH_2_PO_4_•2H_2_O, 3.3 g/l (NH_4_)_2_SO_4_, 0.26 g/l MgCl_2_•6H_2_O, and 2 g/l yeast extract. To investigate the effect of CO_2_ on bacterial growth, 1 g/l NaHCO_3_ was added to the medium.

### Construction of PDHC-Deficient Mutant and Other Mutants

The fragment containing *aceE*, *aceF*, and *lpdA* was deleted for construction of PDHC-deficient mutant. In addition, the fragment containing *pflA* and *pflB* was deleted for construction of *pflA*-*pflB* mutant, and the fragment containing *fdnG*, *fdnH*, and *fdnI* was deleted for construction of *fdnG*-*fdnH*-*fdnI* mutant. All the mutants were derived from the parent strain *K. pneumoniae* KG2 by using lambda Red recombination [20]. The fragment disruption cassettes with a kanamycin resistance marker and flippase recognition site were amplified by two-step PCR from the chromosomal DNA of *K. pneumoniae* KG2 and pKD4 vector, respectively, using the primers listed in Table 1. For the construction of PDHC-deficient mutant, pKD46-Tc was first transformed into *K. pneumoniae* KG2, followed by transformation with the fragment-PD disruption cassette. The recombinant strain was selected from Luria–Bertani (LB) plates supplemented with kanamycin after a 24-h culture at 37°C. Then, pCP20-Tc was transformed into the recombinant strain and incubated at 37°C to remove the kanamycin resistance gene. Finally, the transformants were diluted and plated onto solid LB medium at 42°C to obtain a PDHC-deficient mutant named KG-PD that was no longer resistant to kanamycin and tetracycline hydrochloride. Similarly, by using the same procedure, *pflA*-*pflB* was deleted from the genome of *K. pneumoniae* KG2 to obtain the mutant KG-PF. The mutant KG-PDN was derived from KG-PD by further deletion of *fdnG*-*fdnH*-*fdnI*. All the mutants derived in this study are listed in Table 1.

### Metabolites Analysis

The optical density (OD) of the bacterial cultures was related to the dry cell weight by an experimentally determined calibration curve. The OD of the bacterial cultures at 620 nm (OD_620_) was measured after appropriate dilution. The concentrations of 1,3-PD, 2,3-BD, ethanol, and acetoin in the fermentation broths were quantified by using gas chromatography. The analysis conditions included N_2_ as the carrier gas, detector temperature of 270°C, and column temperature of 110°C. Other metabolites present in the fermentation broths were quantified using high-performance liquid chromatography system equipped with a 2487 Dual-Wavelength Absorbance Detector (Waters Corporation, USA) and a Plastisol ODS column (AQ-C18, 5 μm, 250 × 4.6 mm; Welch Material, Inc., USA) at a flow rate of 0.6 ml/min and column temperature of 60°C. The mobile phase was 2.5 mM H_2_SO_4_. For the investigation of extracellular metabolites, 1 ml of the culture was centrifuged for 1 min at 12,000 ×*g* in a microcentrifuge and the supernatant was filtered through a 0.45-μm syringe filter before analysis.

### Enzyme Assays and Determination of NAD^+^ and NADH

The activities of PDHC and PFL were assayed using the methods described previously [21, 22]. Crude protein was measured by the method of Bradford with bovine serum albumin as the standard [23]. For anaerobic enzyme assays, strict anoxic condition was employed as described previously [24]. The cells were harvested after 12 h of flask culture, and then washed thrice with cold 50 mM potassium phosphate buffer containing 1 mM dithiothreitol (pH 7.0). The resuspended cells were disrupted by freezing and thawing, and the supernatant was harvested after centrifugation for 20 min at 12,000 ×*g* at 4°C for enzyme detection. One unit (U) of enzyme activity represents the amount of enzyme catalyzing the conversion of 1 μmol of substrate per min into specific products, and U/mg refers to the enzyme activities in 1 mg of crude protein extract. The intracellular concentrations of NAD^+^ and NADH were determined using the procedures described by Cui *et al*. [12].

## Results

### Growth of the PDHC-Deficient Mutant

**Fig. 2:**
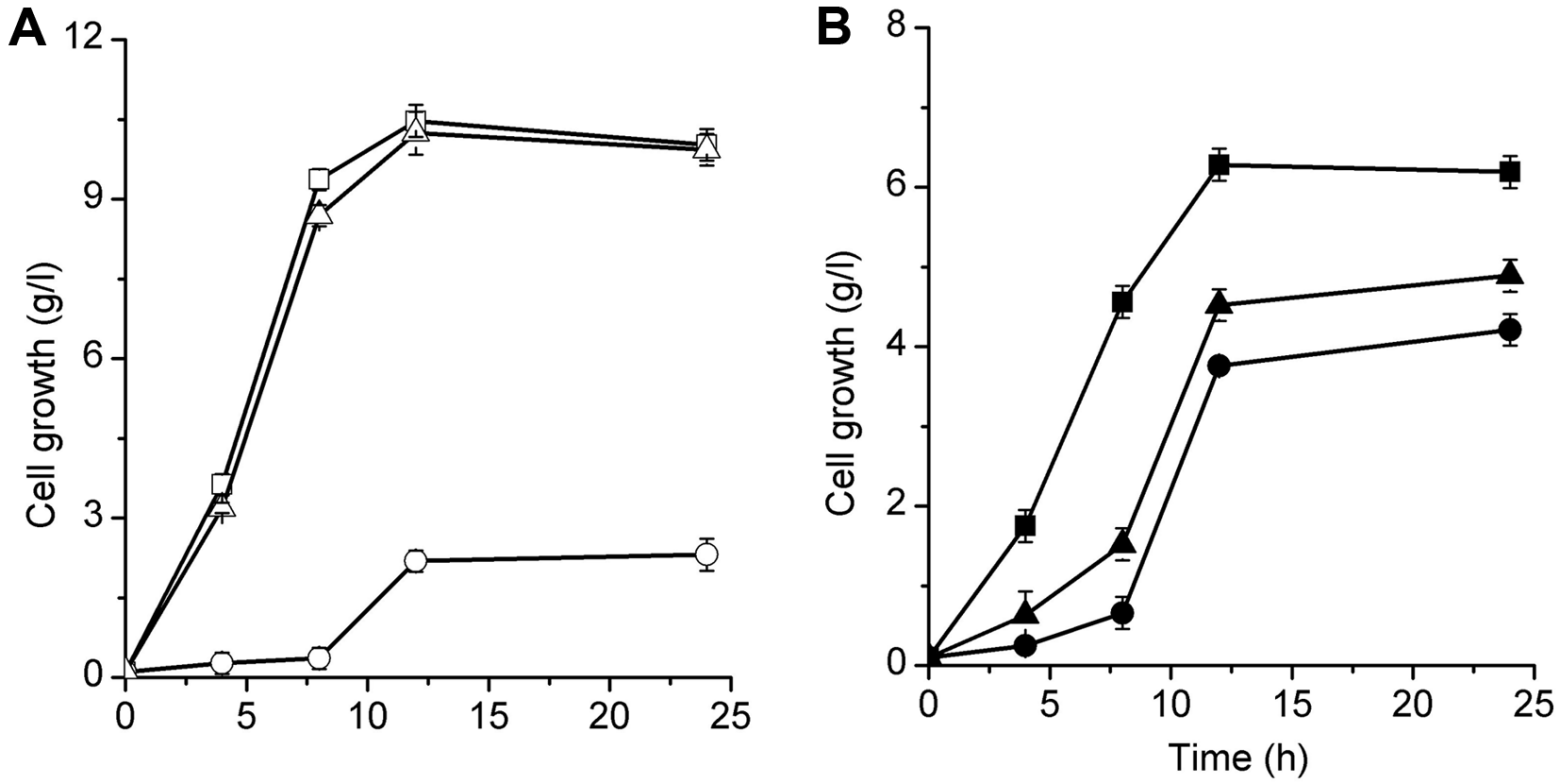
Cell growth of KG2 (*square*), KG-PD (*circle*) and KG-PF (*triangle*) during a 24 h batch flask cultivation under aerobic (*empty symbol*) and anaerobic (*solid symbol*) conditions. Date points are average of three identical experiments.

PDHC contains three enzymatic components: E1, E2, and E3, which are encoded by *aceE*, *aceF*, and *lpdA*, respectively [25]. In the present study, these three contiguous genes were deleted for the construction of the PDHC-deficient mutant, KG-PD. The growth of KG-PD during batch culture in glycerol-based medium is shown in Fig. 2. As aerobic catabolism of pyruvate to AcCoA has been reported to be mainly dependent on PDHC [26], as expected, the aerobic growth of KG-PD was severely inhibited, with cell concentration decreasing to 2.31 g/l, which was only 23% of that of the parent strain (Fig. 2A) at the end of the culture period. Interestingly, KG-PD exhibited growth defect under anaerobic conditions as well, with the cell concentration decreasing to 4.21 g/l after 24 h of culture, which was 68 % of that of *K. pneumoniae* KG2 (Fig. 2B).

It has been reported that under anaerobic condition, the catabolism of pyruvate to AcCoA is mainly dependent on PFL [27]. The growth of the *pflA*-*pflB* mutant, KG-PF, is shown in Fig. 2. As expected, the aerobic growth of KG-PF was almost the same as that of *K. pneumoniae* KG2, whereas a growth defect of KG-PF was observed under anaerobic condition. However, unexpectedly, the anaerobic growth of KG-PF was better than that of KG-PD. Thus, these findings strongly indicated that PDHC was not only required for aerobic but also for anaerobic catabolism of pyruvate to AcCoA in *K. pneumoniae*.

### Analysis of the Metabolites and Enzymes of the PDHC-Deficient Mutant

The metabolites of the PDHC-deficient mutant, KG-PD, determined after 24 h of batch culture are listed in Table 2. As the parent stain is a lactate-deficient 1,3-PD producer, the 1,3-PD level reached 10.2 and 18.5 g/l at the end of aerobic and anaerobic culture, respectively. While formate was almost undetected, the other three byproducts originating from pyruvate could be detected: 2,3-BD was the main byproduct and its accumulation was higher during aerobic culture (6.12 g/l), whereas the accumulation of acetate and ethanol was higher during anaerobic culture (1.44 and 3.52 g/l, respectively, at the end of batch culture with *K. pneumoniae* KG2). Furthermore, high concentration of acetoin, the precursor of 2,3-BD, was noted (2.12 g/l) in *K. pneumoniae* KG2 under aerobic condition.

When compared with *K. pneumoniae* KG2, the PDHC-deficient mutant, KG-PD, exhibited high accumulation of pyruvate (3.98 g/l) under aerobic condition. However, under anaerobic condition, no accumulation of pyruvate in KG-PD was noted, when compared with that in *K. pneumoniae* KG2. With regard to the other metabolites (1,3-PD, 2,3-BD, and ethanol), their accumulation was extremely low in KG-PD under both aerobic and anaerobic conditions, while the accumulation of acetate slightly increased during anaerobic cultivation. Table 2 presents the accumulation of metabolites in the *pflA*-*pflB* mutant, KG-PF, after 24 h of flask culture. It can be noted from the table that under both aerobic and anaerobic conditions, pyruvate was not accumulated, whereas the accumulation of 1,3-PD and 2,3-BD increased and that of ethanol decreased.

**Fig. 3:**
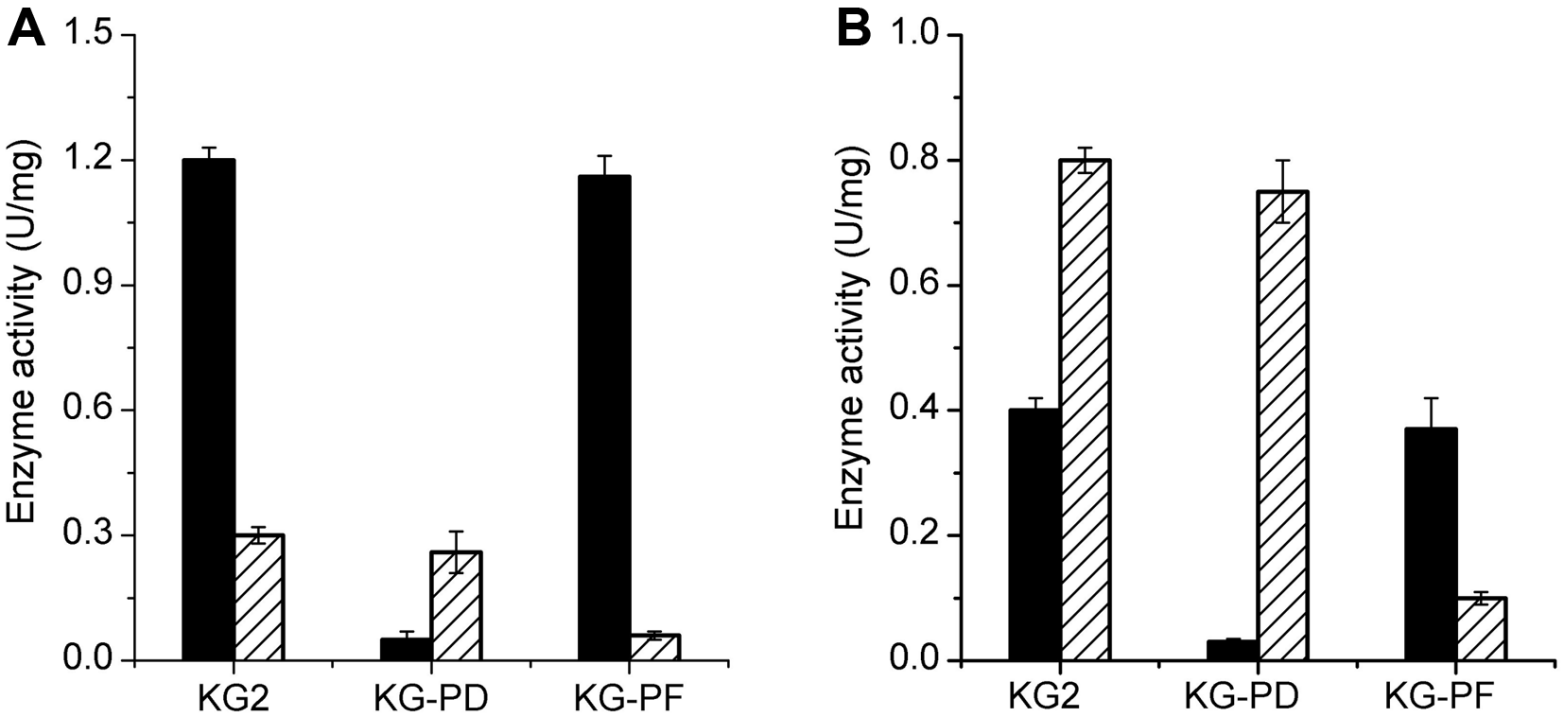
The pyruvate dehydrogenase complex and pyruvate formate lyase activities of parent strain KG2 and mutant strains of KG-PD and KG-PF under aerobic condition (a) and anaerobic condition (b) after 12 h batch flask cultivation. Black bars represent PDHC activity while hatched bars represent PFL activity. Error bars represent the standard deviations from three independent experiments.

The activities of PDHC and PFL in different strains after 12 h of flask culture are shown in Fig. 3. Under aerobic condition, the PDHC activity in the parent strain *K. pneumoniae* KG2 was 1.20 U/mg, which was nearly fourfold higher than that of PFL (Fig. 3A). However, in KG-PD, the activity of PDHC was completely eliminated, combined with severe growth defect and high accumulation of pyruvate, clearly suggesting that PDHC is responsible for the generation of carbon flux to AcCoA needed for cell growth under aerobic condition. During anaerobic cultivation, the carbon flux to AcCoA in *K. pneumoniae* KG2 appeared to mainly depend on PFL, with its activity reaching 0.81 U/mg, which was nearly twofold higher than that of PDHC. Accordingly, in KG-PF, extremely low PFL activity (Fig. 3B) was accompanied by severe anaerobic growth defect caused by the lack of carbon flux to AcCoA. However, more severe anaerobic growth defect (Fig. 2B) was noted in KG-PD. Thus, the role of PDHC in anaerobic cultivation of K. pneumonia may not be limited to the generation of carbon flux to AcCoA.

**Fig. 4:**
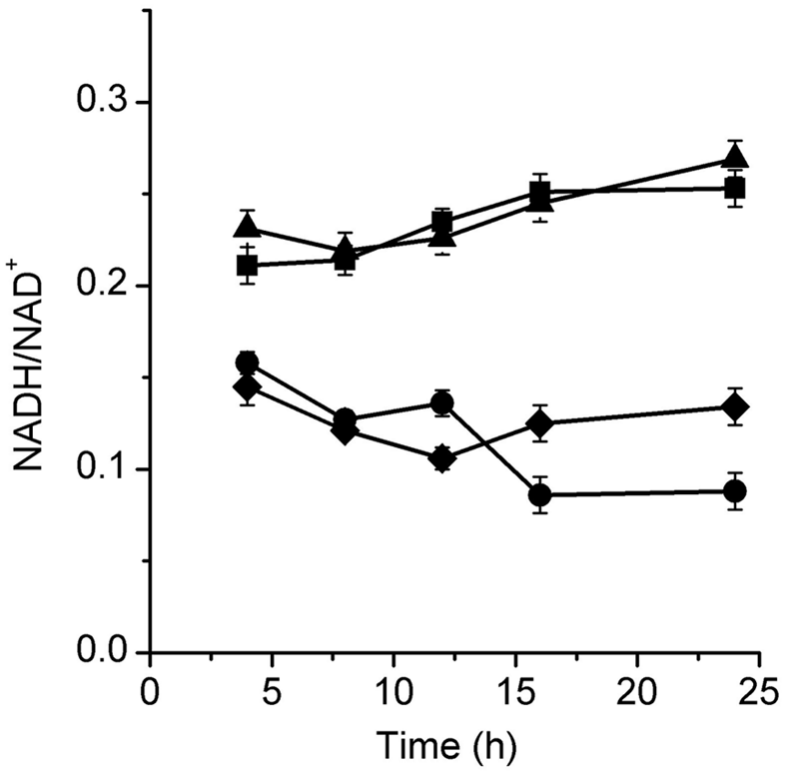
Intracellular redox balance (NADH/NAD^+^) profiles during anaerobic flask culture of KG2 (*square*), KG-PD (*circle*), KG-PF (*triangle*), and KG-PDN (*rhombus*). Date points are averages of at least three identic experiments.

When compared with PFL, pyruvate catalyzed by PDHC generates NADH, which could affect the redox status of the cells. The redox status of strains was analyzed. The ratios of NADH/NAD^+^ in mutants under aerobic condition maintained the same level as that in the parent strain KG2 (data no shown), and the variations in NADH/NAD^+^ in strains under anaerobic condition are shown in Fig. 4. In the parent strain *K. pneumoniae* KG2 and the *pflA*-*pflB* mutant, KG-PF, the NADH:NAD^+^ ratios were around 0.20–0.25 and about 0.25 at the end of anaerobic culture. However, in the PDHC-deficient strain, KG-PD, the redox balance was disrupted, with the NADH:NAD^+^ ratio decreasing to 0.09 at the end of the anaerobic culture, which was only 35 % of that in *K. pneumoniae* KG2.

### Further Deletion of *fdnG*-*fdnH*-*fdnI* in the PDHC-Deficient Mutant

**Fig. 5:**
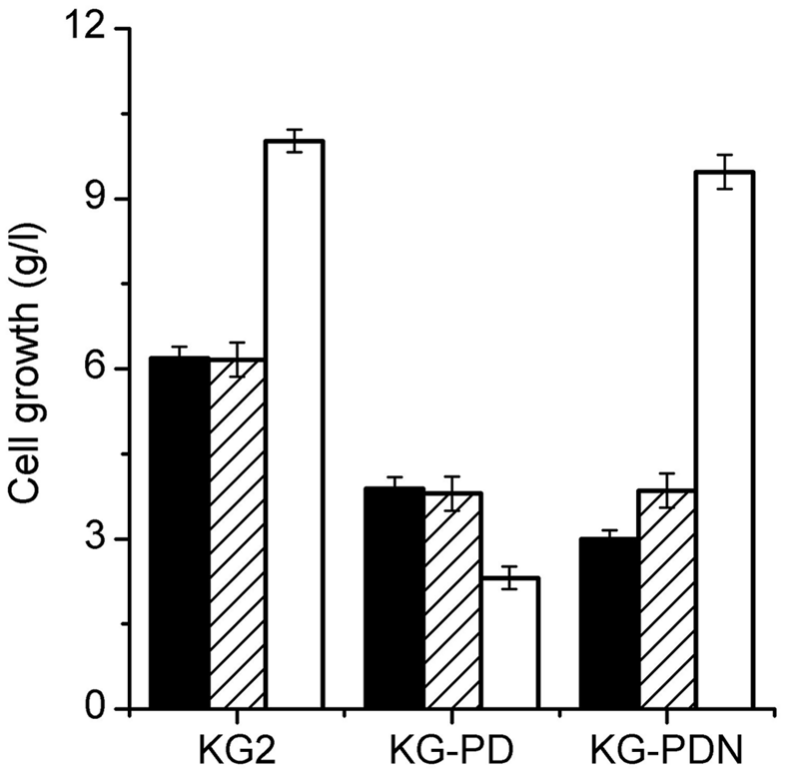
The cell growth of KG2, KG-PD and KG-PDN at the end of flask cultivation. Black bars without NaHCO_3_, hatched bars with NaHCO_3_, both were cultured under anaerobic condition. Empty bars represent cell growth without NaHCO_3_ under aerobic condition. Error bars represent the standard deviations from three independent experiments.

Besides generating carbon flux to AcCoA and NADH, pyruvate catabolized by PDHC also generates CO_2_. It has been reported that the generation of CO_2_ by PDHC is required for the anaerobic growth of *E. coli* [17]. To investigate the effect of CO_2_ on the anaerobic growth of KG-PD, 1 g/l NaHCO_3_, an efficient source of CO_2_ for bacterial growth [28, 29], was added to the culture medium. As shown in Fig. 5, anaerobic growth was not promoted by the addition of NaHCO_3_. It has been demonstrated that the conversion of pyruvate to acetate catalyzed by pyruvate oxidase (PoxB) in *K. pneumoniae* can generate CO_2_, which can effectively provide endogenous CO_2_ for anaerobic growth [10]. In the present study, we found that more acetate was produced in KG-PD during anaerobic cultivation (Table 2).

As the reaction of formate catalyzed by formate dehydrogenase (FDH) is also an effective mode of endogenous CO_2_ generation [30], the effect of blocking of FDH on the PDHC-deficient mutant was further analyzed. Three kinds of FDHs have been reported, namely, FdnGHI (FDH-N) induced by anaerobiosis and nitrate, FdoGHI (FDH-O) synthesized in the presence of oxygen, and FdhF (FDH-H) synthesized at optimal level during fermentative growth of *E. coli* [31, 32]. All these three FDHs were respectively blocked in the PDHC-deficient mutant; however, only blocking of FdnGHI, encoded by *fdnG*-*fdnH*-*fdnI*, showed effects on the PDHC-deficient mutant under our experimental conditions (data not shown). Under anaerobic conditions, blocking of FdnGHI resulted in further growth defect of the mutant KG-PDN. When compared with KG-PD, the cell concentration of KG-PDN decreased from 3.89 to 3.21 g/l (Fig. 5) at the end of anaerobic cultivation, and the production of acetate increased and reached 2.34 g/l (Table 2). However, when the culture medium was supplied with NaHCO_3_, the anaerobic growth of KG-PDN recovered and reached the same level as that of KG-PD (Fig. 5). Figure 4 also shows the redox status in KG-PDN. A further blocking of FdnGHI appeared to result in better redox status, and at the later stage (after 15 h) of anaerobic growth of KG-PDN, the NADH/NAD+ ratio remained at around 0.15, which was higher than that in KG-PD (around 0.09). Thus, these findings effectively confirmed the presence of sufficient internal CO_2_ pool to meet the anaerobic growth of the PDHC-deficient mutant, KG-PD.

Interestingly, the severe aerobic growth defect noted in KG-PD was completely recovered by further blocking of FdnGHI, and at the end of flask culture under aerobic condition, the cell growth of KG-PDN reached 9.93 g/l, which was almost the same as that of the parent strain. Besides, excessive accumulation of pyruvate disappeared, and when compared with those in the parent strain KG2, high concentrations of acetoin and 3-HP were formed in KG-PDN during aerobic cultivation, which reached 9.5 and 3.57 g/l, respectively, at the end of cultivation (Table 2).

## Discussion

For bioconversion of glycerol to 1,3-PD by *K. pneumoniae*, cell metabolism in pyruvate node should be carefully controlled [33]. The PDHC occupies the key position in the oxidation of pyruvate to AcCoA. However, to date, only few studies have focused on the role of PDHC in *K. pneumoniae*, especially under anaerobic conditions. In the present study, the cell growth and metabolites of PDHC-deficient mutant were analyzed and compared with other strains, and the role of PDHC in *K. pneumoniae* was examined.

Under aerobic condition, pyruvate is mainly converted to AcCoA by PDHC in *K. pneumoniae*, and blocking of PDHC results in high accumulation of pyruvate and severe growth defect. Although PFL has been reported to function during aerobic cultivation of *E. coli* [34], the data of the present study indicated that the carbon flux to AcCoA required for aerobic growth of K. pneumonia seemed to be completely used by PDHC alone, and to balance the extra NADH, the *pflA*-*pflB* mutant secreted more 1,3-PD and 2,3-BD. However, interestingly, the level of ethanol in the *pflA*-*pflB* mutant severely decreased to undetectable level, which should in fact increase because conversion of one molecule of AcCoA to ethanol needs two molecules of NADH, which might be more favorable for the cells to balance the extra NADH, when compared with secretion of 1,3-PD or 2,3-BD [35]. The probable reason for this observation could be that all of the AcCoA molecules in the *pflA*-*pflB* mutant were used for cell growth, resulting in no extra AcCoA for ethanol formation. Thus, in addition to the function of maintaining the cell redox status [36], ethanol seems to be an indicator of AcCoA pool required for cell growth in *K. pneumoniae*. Under anaerobic conditions, pyruvate is mainly converted to AcCoA by PFL. However, blocking of PDHC results in more severe growth inhibition, when compared with that of PFL, indicating that the role of PDHC may not only be limited to the generation of carbon flux to AcCoA during anaerobic cultivation of *K. pneumoniae*. It has been reported that the critical role of PDHC is to generate CO_2_ needed for the anaerobic growth of *E. coli*. However, in the present study, addition of extra CO_2_ did not promote cell growth and caused an imbalance in the redox status in the PDHC-deficient mutant, thus strongly indicating that the critical role of PDHC is generation of NADH and not CO_2_ for anaerobic growth of *K. pneumoniae*. Further studies are needed to explore the reason for the anaerobic growth of *K. pneumoniae* using excess NADH, which could be an important feature explaining the ability of *K. pneumoniae* to produce high amount of 1,3-PD. The findings of the present study also showed that *K. pneumoniae* can balance the extra NADH to produce more 1,3-PD by blocking PFL during anaerobic cultivation. Moreover, despite the anaerobic growth defect, highest 1,3-PD production was noted in the *pflA*-*pflB* mutant. In previous studies on *E. coli* and *K. pneumoniae*, anaerobic growth defects of *pflB* mutants have been reported, which were presumed to be owing to the redox imbalance caused by ineffective regeneration of NAD^+^ [34, 37, 38]. However, in the present study, combined with the balanced redox status and low level of ethanol, the lack of AcCoA pool could be the reason for the anaerobic growth defect of the *pflA*-*pflB* mutant. Thus, an improvement in the carbon flux for fixing the growth defect of the *pflA*-*pflB* mutant could be an effective strategy for constructing the high-yielding 1,3-PD-producing *K. pneumoniae* strain.

Unlike PDHC, there could be several PFLs in the cells, and it has been reported that PFL-I (encoded by *pflB* and *pflA*) is dominant in *E. coli* [39]. Similarly, in the present study, deletion of *pflA*-*pflB* severely blocked the activities of PFL in *K. pneumoniae*. PFL catalyzes the conversion of pyruvate to AcCoA and formate. Formate is an important electron donor, which is oxidized by FDH to generate CO_2_. In the PDHC-deficient mutant, almost no accumulation of formate was noted, suggesting high activity of FDH in *K. pneumoniae*. Therefore, the effect of blocking of FDH on the PDHC-deficient mutant was further analyzed. Among the three kinds of FDHs blocked, only blocking of FdnGHI showed the effects on PDHC-deficient *K. pneumoniae* under our experimental conditions.

Under anaerobic conditions, further blocking of FdnGHI in the PDHC-deficient mutant resulted in a decrease in cell growth, which was recovered by the addition of CO_2_, indicating the lack of CO_2_ in the FdnGHI/PDHC double-deficient mutant. Furthermore, sufficient CO_2_ pool for anaerobic growth in the PDHC-deficient mutant was confirmed. Thus, combined with the data of acetate production and redox status, it can be concluded that: 1) blocking of PDHC enhances the PoxB pathway in the cells to maintain the CO_2_ level for anaerobic growth of *K. pneumoniae*, and the growth defect in this case was caused by the imbalance of redox status; and 2) blocking of both PDHC and FdnGHI resulted in lack of CO_2_ being the key inhibiting factor and further decrease in anaerobic growth, and a better level of redox status was noted in this case, when compared with that found following blocking of PDHC only.

Interestingly, under aerobic conditions, an unexpected good growth of FdnGHI/PDHC double-deficient mutant was observed. Nevertheless, further studies are required to determine how blocking of FdnGHI promotes aerobic growth of PDHC-deficient mutant. As formate, which is also a carbon source [40], was not accumulated following blocking of FdnGHI, the cells seemed to reutilize formate to provide carbon flux, which might have been acquired from pyruvate catabolized by PDHC, for cell growth in FdnGHI/PDHC double-deficient mutant. Nevertheless, blocking of FdnGHI and PDHC triggered metabolic flux redistribution during aerobic cultivation of *K. pneumoniae*, resulting in high production of 3-HP and acetoin, which could probably be attributed to the lack of PDHC [11, 13]. It should be noted that the FdnGHI/PDHC double-deficient mutant showed the ability for the production acetoin, which is also a valuable platform chemical in recent times [41, 42].

In summary, the role of PDHC in *K. pneumoniae* was analyzed in glycerol-based medium. As expected, under aerobic condition, the carbon flux from pyruvate to AcCoA mainly depended on PDHC, whereas under anaerobic condition, the critical role of PDHC appeared to provide NADH for the balance of redox status. Based on our findings, it can be suggested that an improvement in the carbon flux in the PFL-deficient mutant might be an efficient strategy to construct a high-yielding 1,3-PD-producing *K. pneumoniae*, and that FdnGHI/PDHC double-deficient mutant has the ability to produce acetoin.

## Figures and Tables

**Table 1:** Strains, plasmids, primers used in this study.

Strain, plasmid or primers	Relevant genotype and description	Reference or source
Strains		
*K. pneumoniae* KG2	parent type, Amp^r^ Δ*ldhA*	Zhu *et al*.[11]
*K. pneumoniae* KG-PD	Amp^r^ Δ*ldhA* Δ*aceE*-aceF-*lpdA*	This study
*K. pneumoniae* KG-PF	Amp^r^ Δ*ldhA* Δ*pflA*-*pflB*	This study
*K. pneumoniae* KG-PDN	Amp^r^ Δ*ldhA* Δ*aceE*-aceF-*lpdA* Δ*fdnG*-*fdnH*-*fdnI*	This study
Plasmids		
pKD4	Kan^r^, Amp^r^, ori R6Kgamma, rgnB	This lab
pKD46-Tc	Tc^r^, repA101(ts),oriR101, araBp-gam-bet-exo	This lab
pCP20-Tc	Tc^r^, Cm^r^ , ts-rep, [cI857] (lambda)(ts), FLP	This lab
pMD 18T	Cloning vector, Amp^r^	TAKARA
Primers		
*aceE*-1	5' GAAATGCTCCCGTTGGTC 3'	
*aceE*-2	5' AAGCAGCTCCAGCCTACACATTAA	
GAACCAGAGTTCTCGT 3'		
*lpdA*-3	5' AGGAGGATATTCATATGGACGGCG	
CGATTGTCGGCACC 3'		
*lpdA*-4	5' TTACTTTTTCTTCGCTTTGG 3'	
*pflA*-1	5' ACTTTGTGACCATACT 3'	
*pflA*-2	5' AAGCAGCTCCAGCCTACACAGATG	
ATTCCGCGCACC 3'		
*pflB*-3	5' AGGAGGATATTCATATGGACGCAG	
TGTCAAAATCAACAGG 3'		
*pflB*-4	5' GCCTGGGAAGGTTTTG 3'	
*fdnG*-1	5' CACTGGGTGGATATCA 3'	
*fdnG*-2	5' AAGCAGCTCCAGCCTACACAGTAC	
CGGAACGAATCG 3'		
*fdnI*-3	5' AGGAGGATATTCATATGGACGCCT	
GCTGATTCATGC 3'		
*fdnI*-4	5' CCTTCACGACTCTCTTTCAT 3'	

**Table 2:** Metabolites of KG2, KG-PD, KG-PF, KG-PDN in glycerol-based medium under aerobic and anaerobic conditions at 24 h in shake flask.

	Aerobic				Anaerobic			
	
	KG2	KG-PD	KG-PF	KG-PDN	KG2	KG-PD	KG-PF	KG-PDN
1,3-PD (g/l)	10.2±0.4	2.1±0.3	13.0±0.3	10.4±0.4	18.5±0.4	10.5±0.3	20.3±0.3	10.2±0.4
2,3-BD (g/l)	6.12±0.31	ND^a^	7.22±0.22	2.62±0.31	4.71±0.30	2.32±0.11	6.42±0.12	4.11±0.21
Acetoin (g/l)	2.11±0.20	ND	3.00±0.20	9.51±0.20	ND	ND	ND	ND
Ethanol (g/l)	2.32±0.31	ND	ND	ND	3.52±0.31	1.51±0.22	0.61±0.23	ND
Pyruvate (g/l)	0.04±0.02	3.98±0.02	0.05±0.01	0.04±0.02	0.06±0.01	0.04±0.02	0.06±0.01	0.05±0.02
Formate (g/l)	0.06±0.02	0.08±0.02	ND	0.07±0.02	0.08±0.02	0.06±0.02	ND	0.06±0.02
3-HP (g/l)	0.35±0.05	0.40±0.02	0.21±0.02	3.57±0.03	0.23±0.03	0.45±0.02	0.18±0.03	0.35±0.03
Acetate (g/l)	0.53±0.02	ND	0.48±0.05	0.50±0.04	1.44±0.03	1.72±0.03	1.20±0.02	2.34±0.02
Glycerol consumption(g /L)	53.2±0.4	22.9±0.5	57.2±0.4	53.9±0.6	49.7±0.5	40.4±0.4	50.8±0.5	38.6±0.8

All results are expressed as the mean ± SD of three independent experiments.

^a^ND, not detected.
